# On the Difficulty of Identifying Cognitive Causes

**DOI:** 10.1007/s42113-026-00289-5

**Published:** 2026-04-20

**Authors:** Heinrich R. Liesefeld, James T. Townsend, Markus Janczyk

**Affiliations:** 1 University of Bremen, Bremen, Germany; 2 Indiana University, Bloomington, IN USA

## Abstract

The target article “Illusions of Understanding in the Sciences” by Shiffrin et al. (*Computational Brain & Behavior*, 2026) demonstrates how scientists tend to misinterpret correlations by declaring one of the variables as the cause of the other. In our commentary, we discuss that even under controlled experiments it is typically not possible to conclusively identify causal relationships beyond a superficial level.

Many of the problems discussed in the target article arise because a causal relationship is assumed to “explain” a correlative relationship. But the bedrock of science are experiments, which do not necessarily suffer from the same interpretational problems. If an experimental manipulation of A has an effect on B and if A and B are objectively observable variables (rather than theoretical constructs) and are not misinterpreted in any way (e.g., as construct pure when they in fact confound various variables; exemplified by Liesefeld & Müller, 2020, for set-size effects in visual search), there is no arguing against A being a cause of B. This universal scientific truth, rather than the interpretation of correlations, must be the origin of our thinking about the validity of science. For example, if distractor presence (A) delays search times (B), distractor presence is one of the causes for search times (Liesefeld & Müller, 2019). For engineering purposes, this level of understanding is sufficient and merely the scope of the method is limited because many experiments are not possible (due to practical or ethical reasons). Hence the apparent success of the scientific method, which is typically demonstrated by technical progress. However, whereas this level of understanding was satisfying to some behaviorists, such explanations feel superficial and unsatisfying to cognitive scientists. The cognitive revolution can be interpreted as the realization that manipulatable variation in A is merely a distal cause of observable variation in B and that there must be intervening cognitive causes C_i_ that are more proximal to B (such as attentional capture in the example; see Liesefeld et al., 2024). Unfortunately, these causes are not directly manipulatable. Even if some C_i_’s were observable, this would not suffice for confirming them as causes of B; we would still merely measure effects of A on B and A on C_i_. There is no way to prove a causal relationship from C_i_ to B as this must remain a correlative relationship without manipulation of C_i_.

Given that every scientist knows that one ought not interpret causality into correlative relationships, why do they still do it time and time again as exemplified in the target article? This erroneous behavior is certainly multi-causal like the studied behaviors themselves. Unawareness of the general induction problem and the need for understanding as discussed in the target article are among the causes. The latter might express as a desire to uncover intervening causes C_i_ and we might think of this desire as scientific curiosity. In the following, we explore how a well-known logical fallacy might contribute to these misinterpretations in light of scientific curiosity.

A cognitive scientist might come up with a model M that predicts behavior B. If M is true, behavior B must show up, or more formally: M ➔ B. Now, if B is indeed observed, many scientists conclude that M is true: [(M ➔ B) & B] ➔ M. This logical fallacy is known as “affirming the consequent” and a common example is to conclude that it has rained from observing a wet street, thus ignoring the many alternative explanations for the wet street. A valid conclusion that looks similar is modus tollens: [(M ➔ B) & ¬B] ➔ ¬M, which is the basis for falsificationism (Popper, 1935). Modus tollens is applied when cognitive scientists contrive and exclude alternative explanations. However, due to the impossibility to directly manipulate proximal cognitive causes and exacerbated by their non-observability, there will always be many alternative explanations that have not yet been considered. Thus, we can only reject cognitive explanations, but we can never accept them based on experimentation (let alone based on a correlation). Also note the inherent asymmetry between the valid rejection of a (cognitive) model and the invalid acceptance of a model: No matter how much valid evidence was collected “in favor” of a model, a single piece of valid evidence disconfirming a model is sufficient for its rejection. This is often overlooked when evidence for and against a model are balanced against each other.

This logical reasoning is further complicated if B cannot be observed directly. In addition to measurement error there is also sampling error in cognitive science: Model predictions (Bs) typically refer to population parameters, such as the population mean, which can only be estimated from a (often highly variable) sample. In other words, our premise that B can be observed, does not hold for cognitive science. This somewhat attenuates the asymmetry between the valid modus tollens and the related fallacy, because both B and ¬B might be concluded erroneously from the sampled data (Townsend, 1975). This is somewhat different from the often-discussed induction problem, because B (the population parameter) is already an abstract construct that cannot be observed directly, but there is also no other instance of B that could be in conflict. Also note that all statistical testing – null-hypothesis significance testing just as Bayesian approaches – relates merely to the consequence or prediction B, but not the theoretical model M or any of B’s unobservable causes C_i_ proper.

To conclude, with the help of experiments (but not by simply observing correlative relationships) and a statistical leap of faith, we can discover that A is one (of potentially many) distal causes of B, but we cannot positively confirm any proximal cognitive cause C_i_ of B. We can find a cognitive model M that is useful to predict B, but with this approach we cannot know whether this model is anyhow related to the truth. Science can progress only by excluding invalid models. As the number of potential non-manipulatable and/or non-observable cognitive mechanisms is unknown and potentially huge, a promising approach is to exclude whole classes of models (e.g., Koob et al., 2023; Townsend & Altieri, 2012). If and only if these classes are logically exhaustive, modus tollendo ponens provides some hope to arrive at valid truth claims and thus satisfy scientific curiosity: If either A_1_ or A_2_ are true and A_2_ is not true, A_1_ must be true: (A_1_ v A_2_) & ¬A_2_ ➔ A_1_.

## Data Availability

No datasets were generated or analysed during the current study.
